# Is It Possible to Restrain OER on Simple Carbon Electrodes to Efficiently Electrooxidize Organic Pollutants?

**DOI:** 10.3390/molecules27165203

**Published:** 2022-08-15

**Authors:** Marija Ječmenica Dučić, Danka Aćimović, Branislava Savić, Lazar Rakočević, Marija Simić, Tanja Brdarić, Dragana Vasić Anićijević

**Affiliations:** 1 University of Belgrade, Vinča Institute of Nuclear Sciences-National Institute of the Republic of Serbia, Department of Physical Chemistry, Mike Petrovića Alasa 12-14, 11001 Belgrade, Serbia; 2 University of Belgrade, Vinča Institute of Nuclear Sciences-National Institute of the Republic of Serbia, Department of Atomics Physics, Mike Petrovića Alasa 12-14, 11001 Belgrade, Serbia

**Keywords:** carbon, graphene, DFT calculations, organic pollutants, electrochemical oxidation, nanocomposite anodes, oxygen evolution reaction

## Abstract

This paper presents a comparative analysis of three carbon-based electrodes: bare multiwalled carbon nanotubes (MWCNT), SnO_2_/MWCNT, and PbO_2_/graphene-nanoribbons (PbO_2_/GNR) composites, as anodes for the electrooxidative degradation of Rhodamine B as a model organic pollutant. Anodic electrooxidation of Rhodamine B was performed on all three electrodes, and the decolorization efficiency was found to increase in the order MWCNT < PbO_2_/GNR < SnO_2_/MWCNT. The electrodes were characterized by X-ray photoelectron spectroscopy (XPS) and linear sweep voltammetry (LSV). It was proposed that, in the 0.1 M Na_2_SO_4_ applied as electrolyte, observed decolorization mainly occurs in the interaction of Rhodamine B with OH radical adsorbed on the anode. Finally, the obtained results were complemented with Density Functional Theory (DFT) calculations of OH-radical interaction with appropriate model surfaces: graphene(0001), SnO_2_(001), and PbO_2_(001). It was found that the stabilization of adsorbed OH-radical on metal oxide spots (SnO_2_ or PbO_2_) compared to carbon is responsible for the improved efficiency of composites in the degradation of Rhodamine B. The observed ability of metal oxides to improve the electrooxidative potential of carbon towards organic compounds can be useful in the future design of appropriate anodes.

## 1. Introduction

Electrochemical oxidation of organic pollutants belongs to Advanced oxidation processes (AOPs), a group of methods used to oxidize organic pollutants in water in a universal manner, up to CO_2_ and H_2_O, when conventional technologies are ineffective [1,2]. Electrochemical oxidation (EO) has attracted considerable attention from researchers due to its simplicity, applicability in systems of different compositions and sizes, and environmental compatibility—because it does not require the addition of toxic chemical reagents [3]. On the other hand, the main stumbling block in the way of the wide use of EO technology remains the high energy consumption and accordingly low effectiveness of the process [4]. Currently, a controlled design of novel, highly specific, highly efficient, and easily synthesized electrode materials, is among the major strategies to improve the cost/efficiency ratio of the process and its applicability in practice [5,6].

Oxygen evolution reaction (OER) [7] has been considered one of the crucial electrochemical processes in technologies for wastewater treatment, since it is fundamentally connected with the efficiency of electrooxidative depollution. Namely, it is competing with electrochemical oxidation of organic pollutants in depletion hydroxyl radical (•OH) [8]. The mechanism of EO is based on the electro-generation of adsorbed •OH on the anode surface as oxygen evolution intermediate from water discharge. The reaction of organic molecules with electrogenerated •OH occurs in competition with the side OER resulting in a decrease in the efficiency of the anodic process. Studies [9,10] have shown that the activity of these electrochemically generated •OH is linked to their interaction with the electrode surface and depends on the nature of anode materials. A low oxidation power (“active“) anodes exhibit high electrochemical activity for OER, i.e., anodes that are good catalysts for the OER, such as carbon, graphite, or platinum, exhibit less efficiency for electrochemical oxidation.

On the contrary, the high oxidation power anodes with high overpotential for OER exhibit a low electrochemical activity for the OER. Namely, anodes that are poor catalysts for the OER, have “nonactive” behavior and favor the total mineralization of the organic compounds to CO_2_ and H_2_O which indicates them as ideal electrodes for wastewater treatment. Although “active“ by nature of OER [11], carbon-based anodes are still interesting for the electrochemical depollution, mostly due to: the boundary overpotential for OER of about 1.7–1.8 V, low price, and good availability from sustainable sources [12], and the high active surface.

Current anode materials that are investigated for electrooxidation of organic pollutants mainly involve noble and transition metals/metal oxides (IrO_2_, RuO_2_, PbO_2_, TiO_2_, SnO_2_) [13,14,15,16,17]. Their high efficiency for depollution is based on the high OER overpotential. On the other hand, research interest to use carbonaceous materials as anodes has been increasing in recent years [18,19]. The development of advanced, high-surface carbonaceous materials opens novel possibilities for the improvement of electrochemical performance. To date, the boron-dopped diamond (BDD) electrode represents a gold standard of efficiency of organic pollutant degradation [20,21,22,23], despite its high cost and limited availability, and there is a growing need for novel, more available materials, with approximate performances.

In this study, we have comparatively investigated decolorization of Rhodamine B dye, as a model pollutant, on three carbon-based anodes: multiwalled carbon nanotubes on stainless steel support (MWCNT@SS), nanocomposite of SnO_2_ and MWCNT (SnO_2_/MWCNT@SS) and nanocomposite of PbO_2_ and graphene nanoribbons on stainless steel support (PbO_2_/GNR@SS). The aim was to provide a systematic insight into the efficiency of combined active/non-active composite electrodes. As shown within the study, the addition of non-active material had a significant impact on the improvement of the degradation rate. Subsequently, the system was modeled by DFT calculations and the principles behind the observed results were clarified. To our knowledge, this is the first study considering principles beyond the competition of OER and OH-mediated electrooxidation of pollutants on composite materials.

## 2. Results

### 2.1. XPS Characterization of the Electrode Coatings

**Figure 1:**
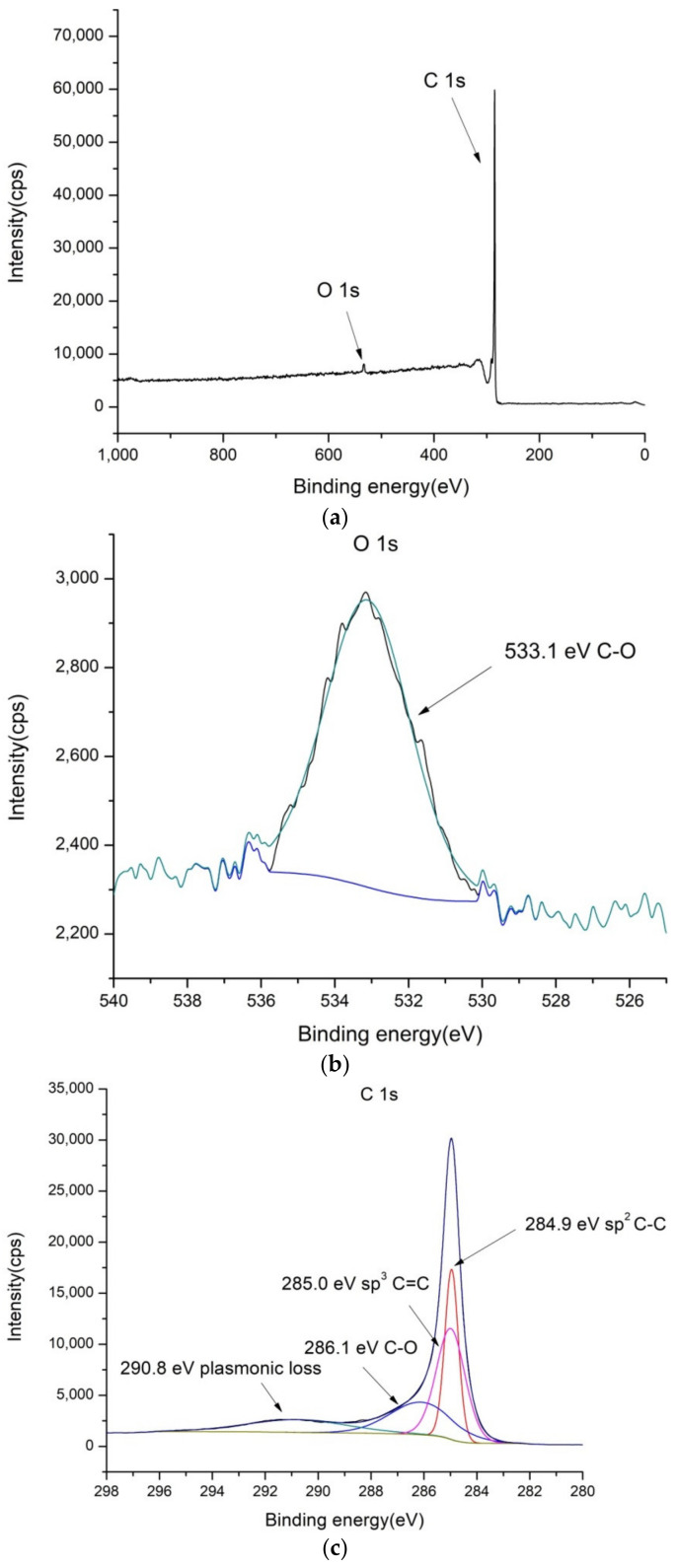
XPS spectra of bare MWCNT: (**a**) low-resolution spectra, (**b**) high-resolution O 1s and (**c**) high-resolution C 1s spectra.

**Figure 2:**
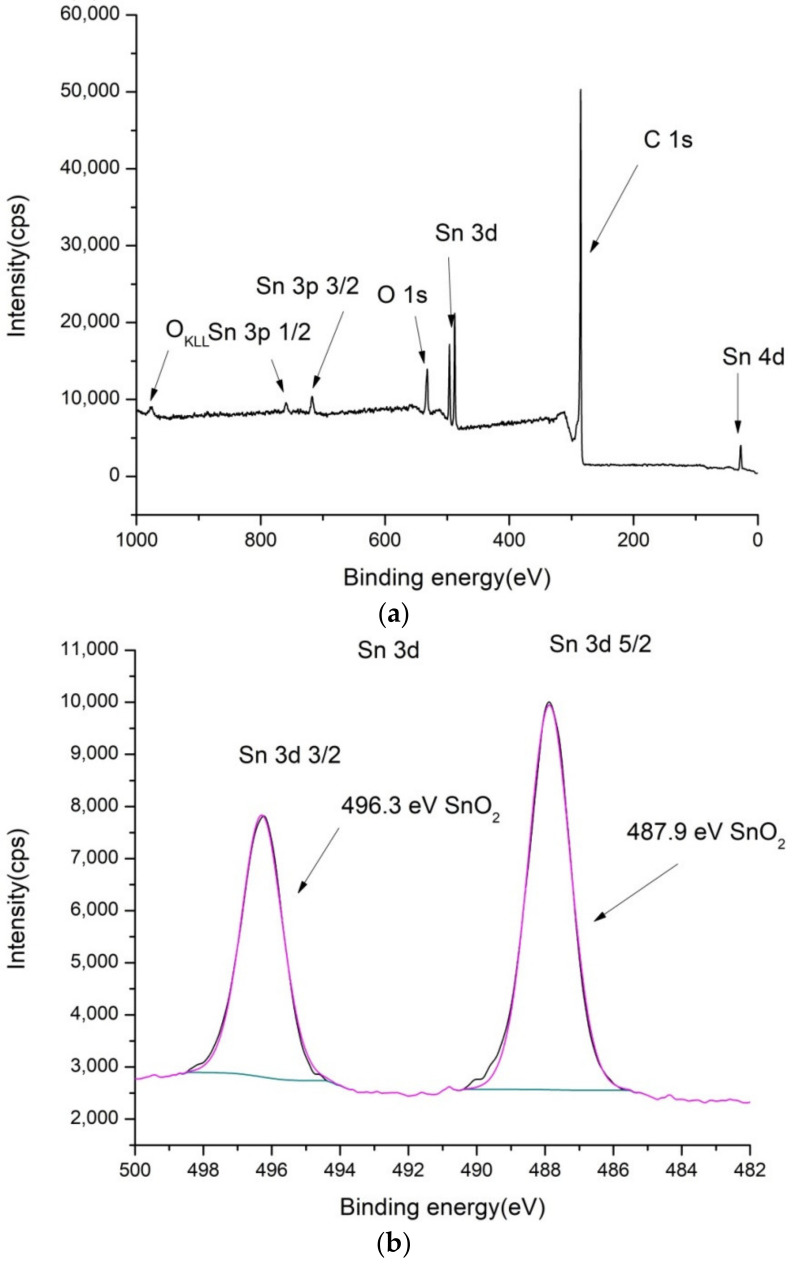

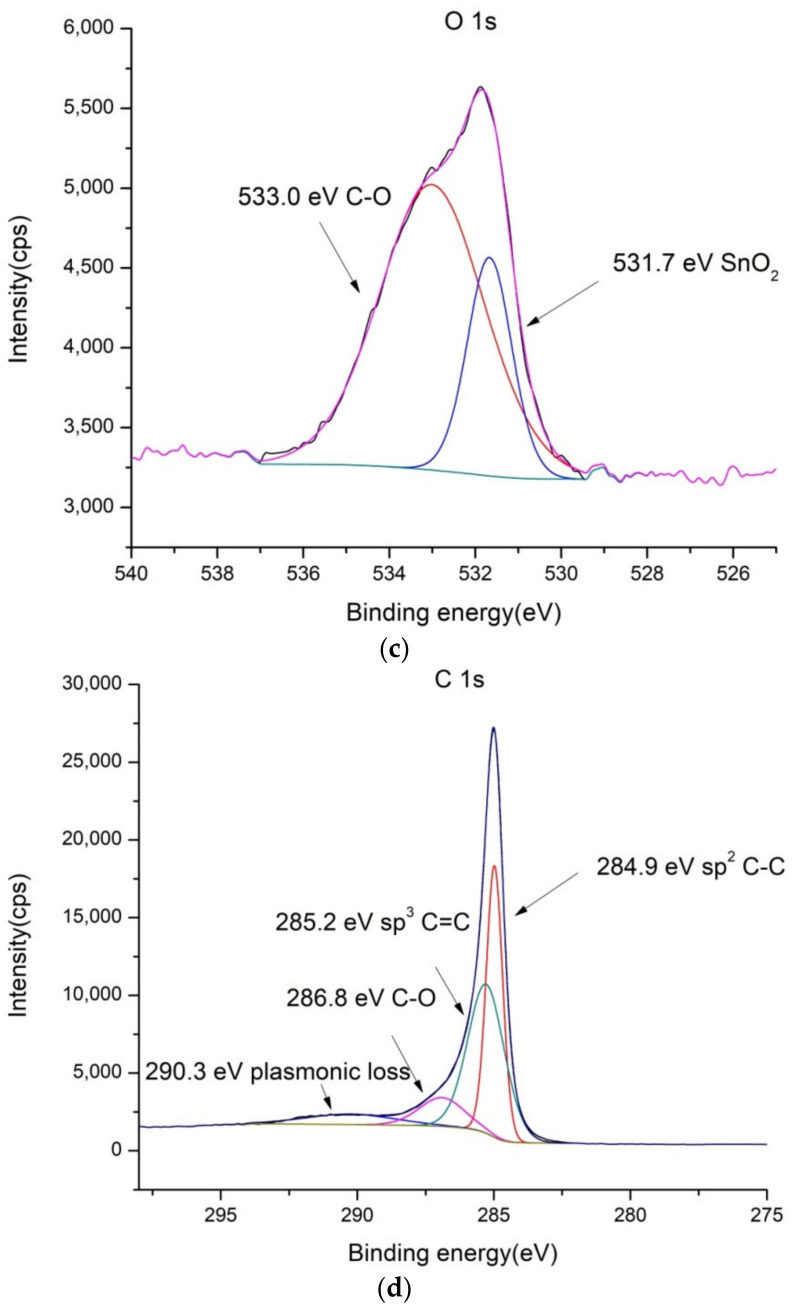
XPS spectra of SnO_2_/MWCNT: (**a**) low-resolution spectra, (**b**) high-resolution Sn 3d, (**c**) high-resolution O 1s and (**d**) high-resolution C 1s spectra.

**Figure 3:**
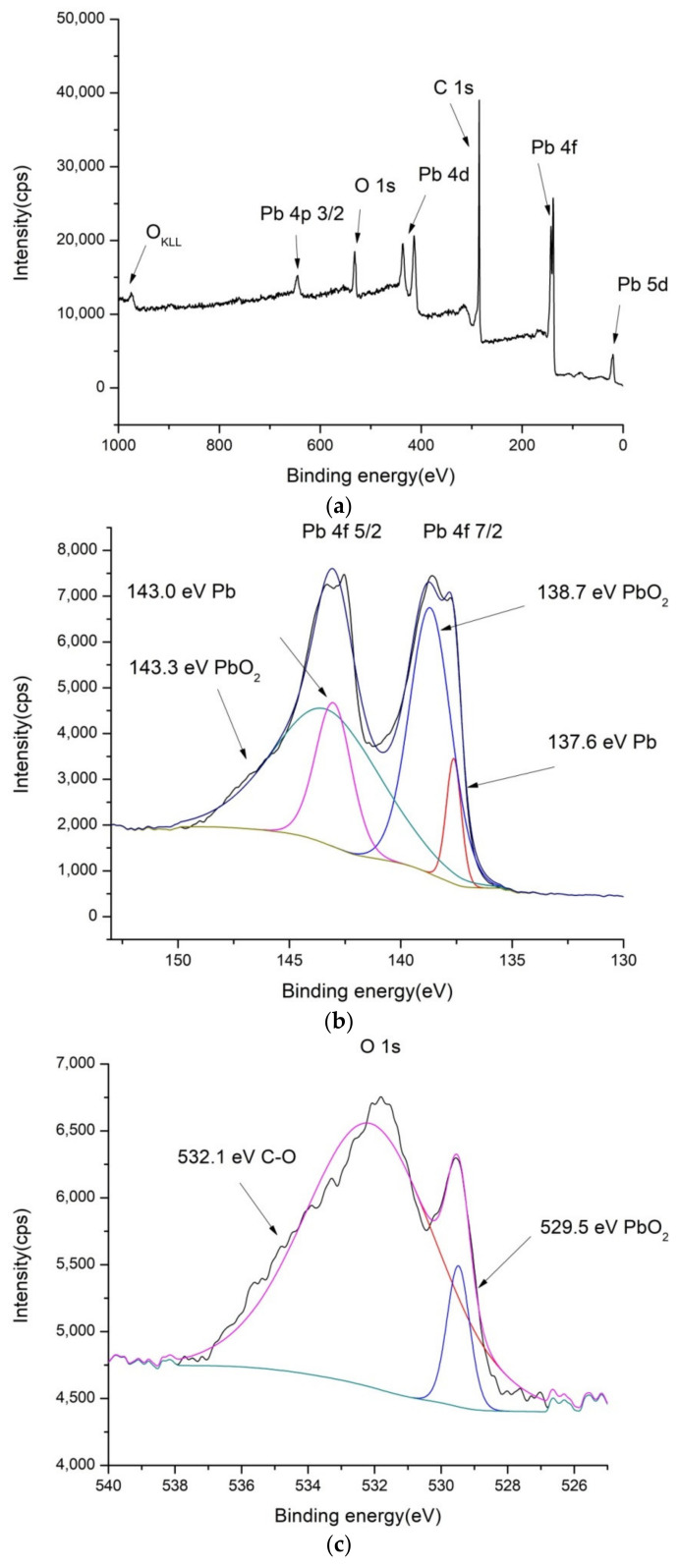

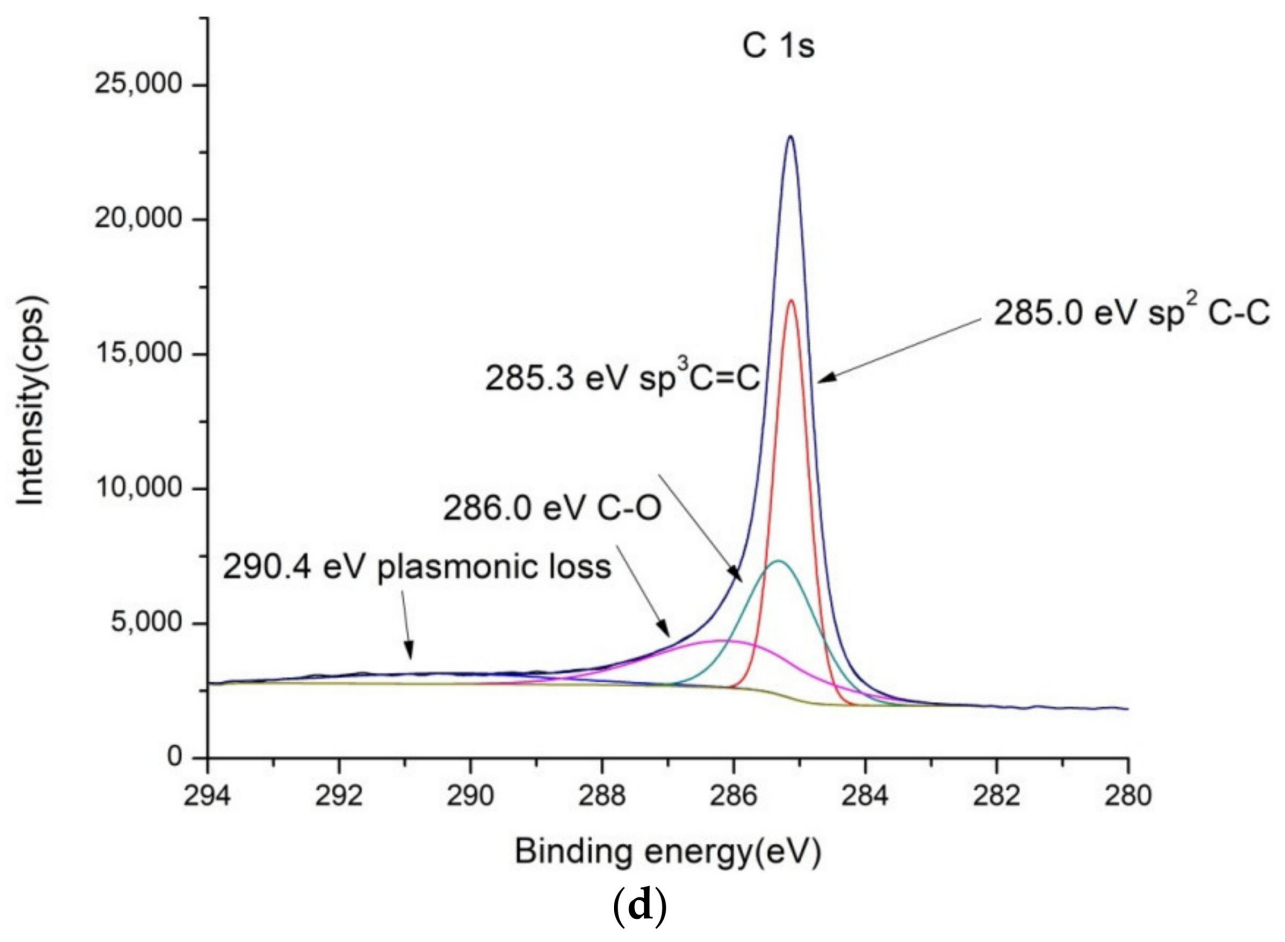
XPS spectra of PbO_2_/GNR: (**a**) low-resolution spectra, (**b**) high-resolution Pb 4f, (**c**) high-resolution O 1s and (**d**) high-resolution C 1s spectra.

To confirm the composition of the electrode surface coatings PbO_2_/GNR, SnO_2_/MWCNT and bare MWCNT, XPS spectra were recorded. Results are shown in Figure 1, Figure 2 and Figure 3.

As can be seen from Figure 1, the MWCNT coating exhibits the usual structure of bonds for carbon materials, consisting of C-C and C-O bonds. The sample was found to contain 1.3% of oxygen and 98.7% of carbon atoms (see Table S1).

In the case of SnO_2_/MWCNT, there are separate phases visible in O1s spectra (Figure 2c) ascribed to SnO_2_ and MWCNT. The content of oxygen is somewhat higher compared to pure MWCNT (5.1%). Detected Sn3d (1.1 at.%) was also ascribed to the single SnO_2_ phase.

In the XPS spectrum of PbO_2_/GNR composite, that are shown in Figure 3, there are also separated phases containing PbO_2_ and carbon material. The structure of carbon binding in GNR is similar to SnO_2_/MWCNT and bare MWCNT. However, the contents of metal oxide are higher than in SnO_2_/MWCNT—contents of Pb is 5.99%, and there is a considerably higher amount of oxygen—11.1% of O1s bonds in the composite (Table S1).

### 2.2. Electrochemical Characterization

**Figure 4:**
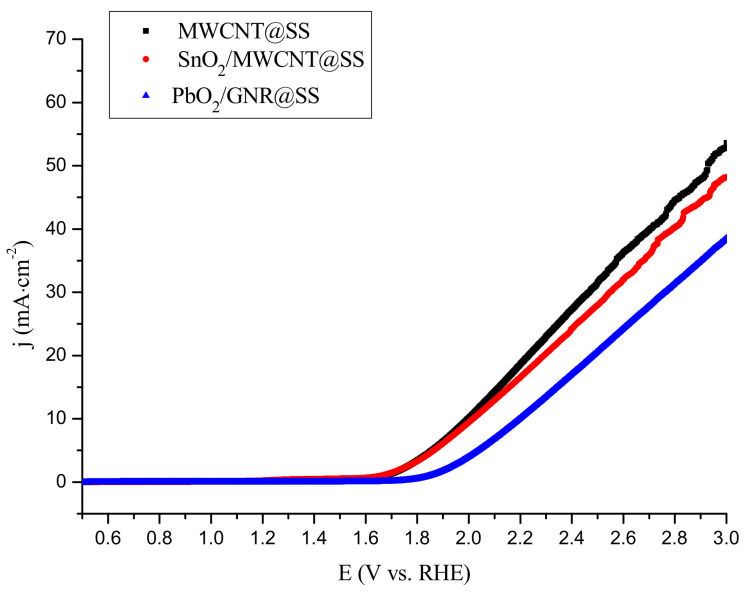
Linear sweep voltammograms of the MWCNT@SS, SnO_2_/MWCNT@SS, and PbO_2_/GNR@SS electrodes in 0.1 mol·L^−1^ Na_2_SO_4_; scan speed 10 mV·s^−1^.

Investigated electrodes were characterized electrochemically in 0.1 M Na_2_SO_4_ as an electrolyte, by Linear Sweep Voltammetry (LSV) technique, at pH 7, at a scan rate of 10 mV·s^−^^1^ (Figure 4).

The current increase observed in LSV diagrams at 1.7–1.8 V vs. RHE originates from OER. As can be seen from Figure 4, the OER onset potentials shift to positive in the order MWCNT@SS ≈ SnO_2_/MWCNT@SS < PbO_2_/GNR@SS. The observed behavior will be discussed in Section 3.

### 2.3. Electrolysis of Bare 0.1 M Na_2_SO_4_ Electrolyte

**Figure 5:**
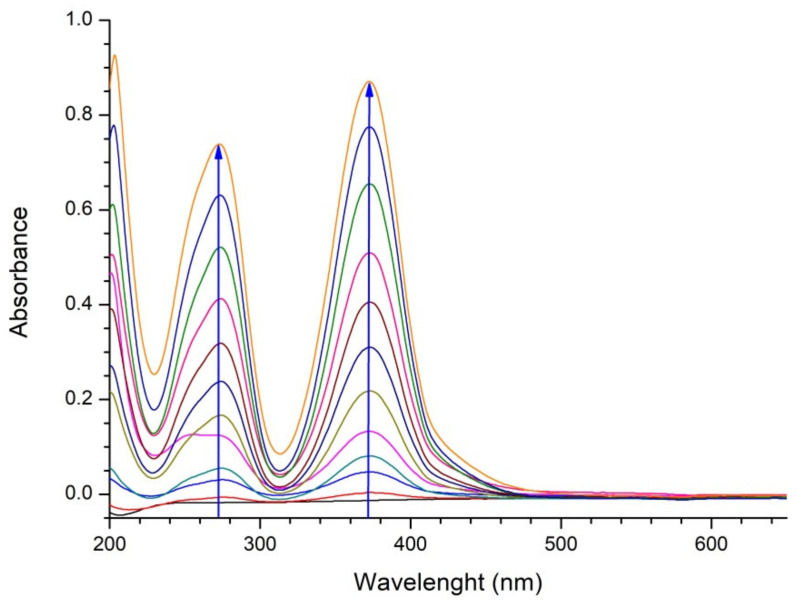
UV-Vis spectra of bare 0.1 M Na_2_SO_4_ electrolyte during 180 min of electrolysis. Direction of evolution of spectra (increase of absorbance) is denoted by arrows.

The bare 0.1 M Na_2_SO_4_ electrolyte was electrolyzed for 180 min, at the MWCNT@SS anode, at a current density of 20 mA cm^−2^. The resulting UV-Vis absorption spectra are represented in Figure 5.

During the electrolysis of bare electrolytes, the colorless Na_2_SO_4_ solution becomes yellow. The increasing maxima at approximately 275 nm and 388 nm probably originate from Fe (III) sulphate, formed as a result of oxidation of iron from SS anode support. However, the observed coloring does not screen the Rhodamine B peak of interest (at 554 nm), as will be confirmed in the following section.

### 2.4. Rhodamine B Degradation

**Figure 6:**
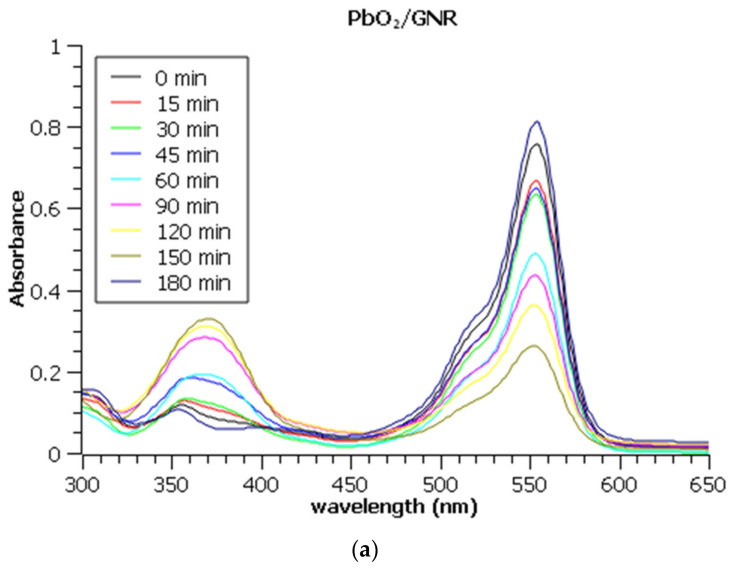

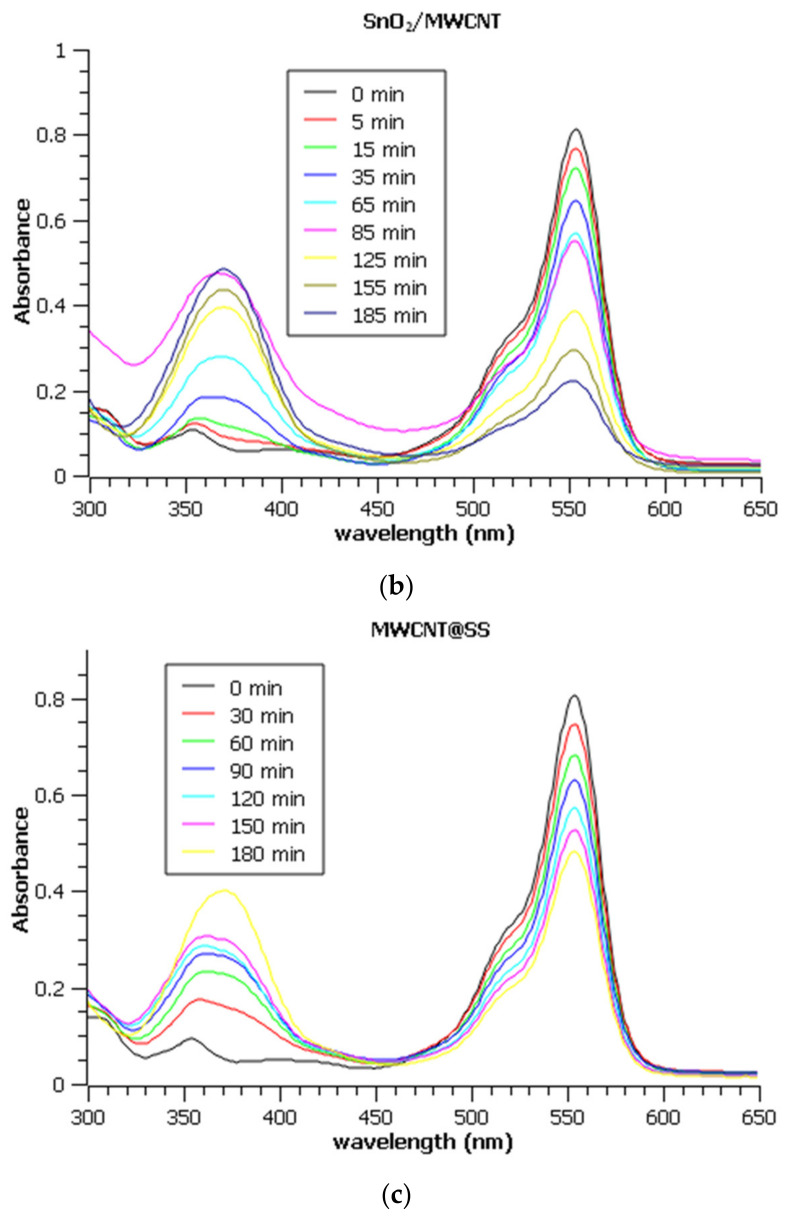
UV Vis absorption spectra of Rhodamine B during electrochemical degradation on (**a**) PbO_2_/GNR@SS, (**b**) SnO_2_/MWCNT@SS, and (**c**) bare MWCNT@SS electrode. Note that all aliquots were diluted 10 times prior to the measurements to assure the applicability of Lambert-Beer’s law.

Absorption spectra of Rhodamine B during 180 min of electrochemical degradation are represented in Figure 6.

The decreasing characteristic absorption band of Rhodamine B is observable in the range of 500–600 nm, with a maximum at 554 nm, while the increasing band between 350 and 400 nm originates from the formation of Fe (III) sulphate.

Degradation kinetics of Rhodamine B was described by a pseudo-first-order equation [24]:

A = A_0_·exp(−k_app_ t) (1)

where A is Rhodamine B absorbance at 554 nm at time (t [min]); A_0_ is initial Rhodamine B absorbance at t = 0 min, k_app_ is apparent (pseudo-first-order) rate constant.

To quantitate decolorization speed, a pseudo-first-order rate constant, k_app_, was calculated from the linearized decolorization curves, according to Equation (2).

ln(A/A_0_) = −k_app_·t (2)

**Figure 7:**
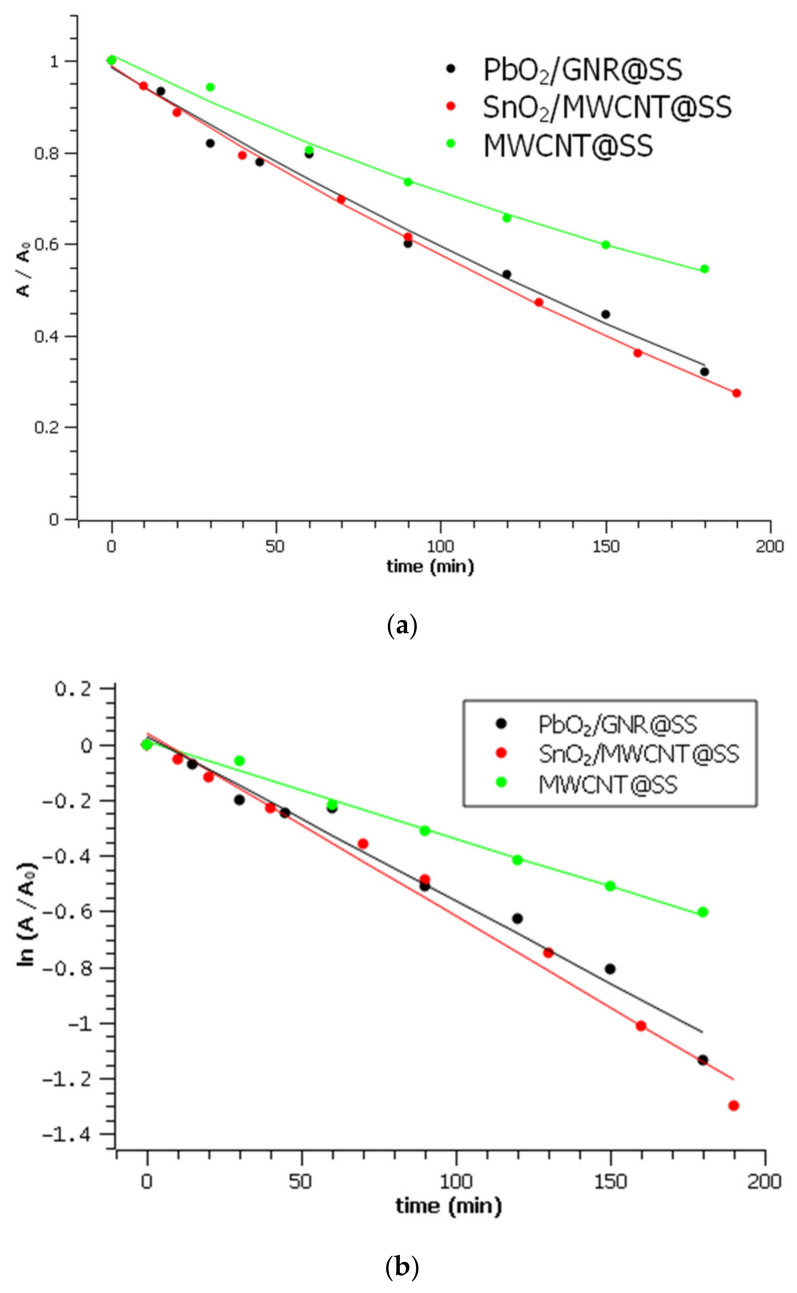
Rhodamine B degradation on three investigated electrodes: (**a**) Degradation curves. A_0_ is the absorbance of Rhodamine B in 0.1 M Na_2_SO_4_ at 554 nm before the electrolysis (**b**) linearized forms of degradation curves with calculated k_app_.

The degradation curves of Rhodamine B on the investigated electrodes, and their linearized forms, are represented in Figure 7.

Within 180 min of electrolysis, more than 70% of Rhodamine B has been decolorized on the most efficient-SnO_2_/MWCNT electrode, while only 40% was decolorized on bare MWCNT for the same time. Calculated pseudo-first-order rate constants are represented in Table 1:

The obtained results generally confirm that the mixing of carbon with metal oxides results in the improvement of electrode efficiency towards the removal of organic pollutants. The most obvious difference in degradation rate was observed between bare MWCNT@SS and SnO_2_/MWCNT@SS.

### 2.5. Role of Hydroxyl Radical in Decolorization of Rhodamine B

The researchers generally agree that the observed decolorization of Rhodamine B in sulphate media most probably arises from the oxidation by OH-radicals generated on the anode [25,26,27,28].

In aqueous solutions, OH-radical is generated in anodic water discharge:

H_2_O + ● → OH● + H^+^ + e^−^ (3)

Here, “●” denotes the adsorption site.

Adsorbed OH radical then interacts with Rhodamine B near the electrode. Alternatively, it is depleted in the formation of adsorbed oxygen (surface oxide), further proceeding to ORR:

OH● → O● + H^+^ + e^−^ (4)

In order to detect atoms of Rhodamine B molecule which are most probable to be attacked by OH-radical, DFT calculations of Fukui reactivity indices [29] were performed.

Fukui index of nucleophilicity represents a tendency of an atom to lose an electron in an electrophilic attack:

f_A_^−^ = q(N) − q(N − 1) (5)

Fukui index of radical attack susceptibility represents reactivity of an atom towards a radical attack:

f_A_^0^ = 1/2·(q(N + 1) − q(N − 1)) (6)

where q(N) is the Bader charge [30] of the N-th Rhodamine B atom.

**Figure 8:**
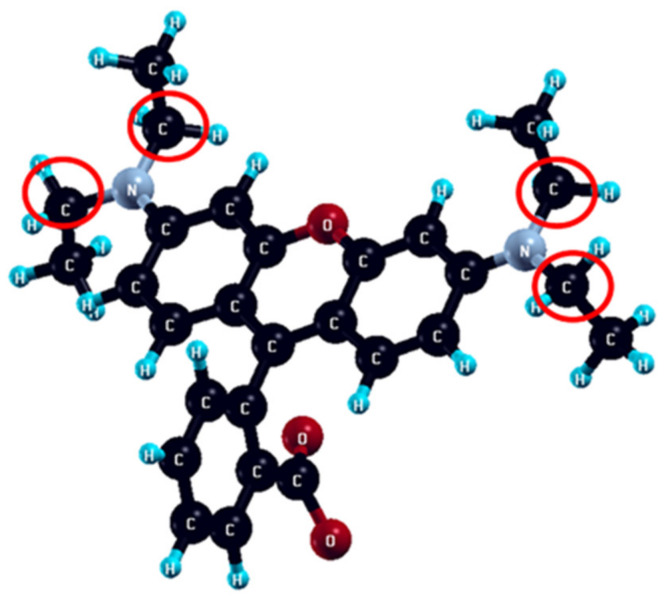
The ethyl atoms of the Rhodamine B molecule most prone to the nucleophilic and radical attacks are denoted by red circles.

Calculated Fukui indices f_A_^−^ and f_A_^0^ for each atom of Rhodamine B molecule are represented in Table S2. Carbon atoms with the most expressed nucleophilicity (f_A_^−^ = 0.24 − 0.25) and radical attack susceptibility (f_A_^0^ = 0.21 − 0.29) are denoted in Figure 8.

### 2.6. DFT Calculations of OH-Radical Formation and Depletion

To understand the interaction of OH radical with electrode material components, the DFT calculations of adsorption of involved species (O and OH) on the model surfaces of interest have been performed. Adsorption energies are represented in Table 2.

As can be seen from Table 2, there is a clear difference in affinity towards the adsorption of O and OH between metal oxides and graphene. Graphene has a rather low affinity for OH-radical compared to PbO_2_ and SnO_2_, but it strongly binds atomic oxygen. SnO_2_ has the highest affinity for OH-radical among the investigated surfaces, but, as well as PbO_2_, it has a considerably lower affinity for the atomic O.

**Figure 9:**
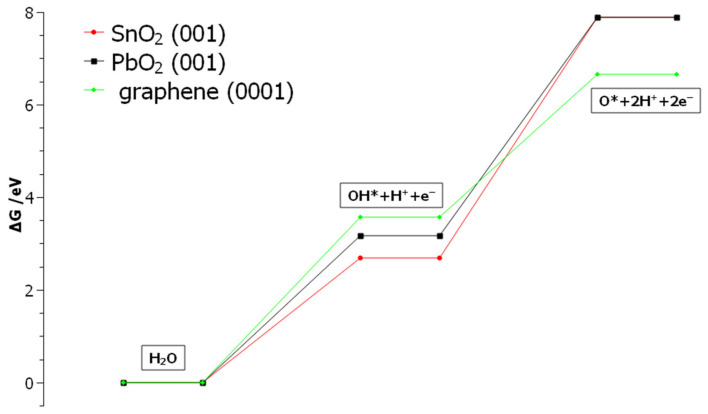
Calculated energetic profiles of OER step 1 (Equation (3)) and step 2 (Equation (4)) at the potential E = 0 V vs. RHE, on the investigated model surfaces graphene(0001), PbO_2_(001), and SnO_2_(001).”OH*” and “O*” denote adsorbed hydroxyl radical and adsorbed atomic oxygen, respectively.

Obtained results are employed to calculate energy profiles of OH-radical formation and depletion on three model surfaces. Starting reactant—isolated water molecule—was taken as an energy zero. Energy profiles for reactions (3) and (4) are represented in Figure 9.

As can be concluded from Figure 9, OH-radical formation requires the least energy at SnO_2_ (followed by PbO_2_), but its further oxidation up to atomic O requires a high amount of energy. Conversely, on graphene OH formation requires the highest amount of energy, but its high affinity for atomic oxygen gives the lowest total energy for surface oxide formation. In summary, OH-radical is more stabilized on the SnO_2_ (followed by PbO_2_) surface, compared to graphene, where it is easily depleted to atomic O and further spent in OER.

## 3. Discussion

In the presented paper, composite carbon-based PbO_2_/GNR@SS and SnO_2_/MWCNT@SS electrodes were prepared, characterized by XPS and LSV techniques, and investigated as anodes for electrooxidative removal of model organic pollutant Rhodamine B.

The LSV characterization of the prepared electrodes was performed to analyze oxygen evolution reaction, as a competitive process to the electrooxidation of organic pollutants. Interestingly, SnO_2_/MWCNT is very similar to bare MWCNT in view of OER behavior, although the measured OER onset value on bare SnO_2_ was above 2.5 V [31]. PbO_2_/GNR exhibits somewhat higher OER potential (1.85 V), which is close to the lower limit of the OER overpotential on bare PbO_2_ (1.8–2.0 V) [28,32]. Obviously, the measured value of the OER overpotential on the composite electrode depends on the contribution of active adsorption sites in each of the two phases. In the case of SnO_2_/MWCNT, where the amount of non-active oxide is low (1.1 at.% Sn as confirmed by XPS), OER completely proceeds on carbon adsorption sites. On PbO_2_/GNR more abundant non-active phases (5.1 at.% Pb) significantly contribute to the measured OER overpotential.

Electrolysis of Rhodamine B was performed in 0.1 M sodium sulfate as an electrolyte, to assure that the degradation will proceed via interaction of Rhodamine B with OH-radical adsorbed on the electrode. Within 180 min of electrolysis, more than 70% of Rhodamine B was degraded on SnO_2_/MWCNT electrode, while only 40% was degraded for the same time on the bare MWCNT electrode. Accordingly, the addition of SnO_2_ and PbO_2_ particles to the carbon electrode materials has improved the degradation of model pollutant Rhodamine B (up to 2-times higher degradation rate compared to bare carbon electrode).

The obtained results are in generally good agreement with the literature data for anodic degradation of Rhodamine B in sulphate media [33,34,35]. The reaction rate can be significantly increased by the use of chloride in electrolyte [18,36,37]. However, indirect oxidation by chlorination is generally less reliable. The use of sulphate electrolyte assures more efficient irreversible degradation [38,39], and more important, avoids the formation of chlorinated by-products which can increase toxicity. Nevertheless, the design of novel electrode materials based on metal oxides [24,40] can significantly contribute to the further improvements of OH-mediated degradation rate, while the advanced, expensive, BDD electrode remains by far the most efficient among the anodes based on carbon.

The nature of the interaction of Rhodamine B with electrogenerated OH-radical, as well as the origin of the observed improvement of degradation efficiency on composite electrodes compared to carbon, was investigated by DFT calculations. For this purpose, Fukui indices of reactivity (f_A_^−^—nucleophilicity index and f_A_^0^—index of susceptibility to radical attack) were calculated for the isolated Rhodamine B molecule.

Obtained results of f_A_^−^ and f_A_^0^ calculations point that the carbon atoms of ethyl groups bound to nitrogen are the most prone to both radical and electrophilic attacks, so the degradation is expected to start from the cleavage of ethyl groups. In this case, it can be proposed that the cleavage disturbs the conjugated system of π-electrons, resulting in discoloration. A similar pathway was confirmed experimentally, for the photocatalytic degradation process, in [41].

The DFT calculations of adsorption energies of O and OH species on model surfaces PbO_2_(001), SnO_2_(001), and graphene(0001) have pointed out that there are differences in the behavior of OH-radical on the graphene and non-active parts of the composite. As confirmed from energy profiles of OH formation and depletion, OH-radical is significantly stabilized on metal-oxide parts of the composite electrode compared to the carbonaceous parts. As a result, on composite electrodes OER is expected to proceed at the same rate on carbon, while metal-oxide parts will play as a reservoir of OH ions, probably being crucial for the improved oxidation rate of Rhodamine B at composites.

The obtained results can be significant for future strategies in the design of composite electrodes for electrochemical depollution. The use of simple carbon electrodes decorated with carefully designed high-surface metal-oxide particles could be a good choice for further advances in the electrooxidation of organic pollutants. Strategically synthesized, high-surface metal-oxide particles will be particularly useful for the improvement of hydroxyl-radical mediated degradation efficiency of organic pollutants on composite materials. On the other hand, investing in advanced high-surface carbons for this purpose will predominantly contribute to the faster formation of higher oxides and increase of OER rate, and thus is not expected to substantially improve the efficiency of electrooxidative degradation of organic pollutants.

## 4. Materials and Methods

### 4.1. Anode Preparation

Nanocomposites PbO_2_/GNR anodes were prepared using a procedure that is explained in detail in our previous paper [42]. The nanosized SnO_2_ was synthesized by the sol-gel method proposed by Kose et al. [43]. A transmission electron microscope (TEM, Talos F200X, FEI Company, Hillsboro, OR, USA) was used to confirm that SnO_2_ particles were obtained at the nano-range scale (Figure S1).

The SnO_2_/MWCNT nanocomposite was obtained as in [31], by mixing synthesized SnO_2_ nanoparticles with commercial MWCNT (particle size 7–15 nm × 3–6 nm × 0.5–200 µm, Sigma-Aldrich, St. Louis, MO, USA), in deionized water, in a ratio 3:1 (*w*/*w*, %) on a magnetic stirrer for 3 h, until a homogeneous suspension was achieved. The sample was dried at 70 °C. After that, obtained SnO_2_/MWCNT nanopowder was dispersed in dimethylformamide (DMF) by sonication for 2 h (5 mg·mL^−1^) and 500 µL suspension was applied dropwise to the stainless steel (SS) electrodes. More details on the characterization of SnO_2_/MWCNT nanocomposite will be provided in our subsequent paper, in preparation [31]. The same dispersion procedure in DMF was applied to commercial MWCNT, and the same volume was dropped to the SS electrode, to obtain the MWCNT@SS electrode. The surface area of the SS electrode was 2 cm^2^ (1 × 2 cm).

### 4.2. X-ray Photoelectron Spectroscopy (XPS)

Synthesized composite samples were analyzed using SPECS Systems with XP50M X-ray source for Focus 500 and PHOIBOS 100/150 analyzer. AlKα source (1486.74 eV) at a 12.5 kV and 32 mA was used for this study. Survey spectra (1000–0 eV binding energy) were recorded with a constant pass energy of 40 eV, step size 0.5 eV, and dwell time of 0.2 s in the FAT mode. Detailed spectra of Pb 4f, Sn 3d, O1s, and C1s peaks were obtained using constant pass energy of 20 eV, step size of 0.1 eV and dwell time of 2 s in the FAT mode. During measurements pressure in the chamber was 1 × 10^−8^ mbar. All the peak positions were referenced to C1s at 284.8 eV. Spectra were collected by SpecsLab data analysis software supplied by the manufacturer and analyzed with commercial CasaXPS software package.

### 4.3. Electrochemical Characterization

Using a Gamry Instrument-Interface 1000 potentiostat/Galvanostat/ZRA 06230, the OER parameters of the electrodes were evaluated by LSV in a conventional three-electrode cell at pH 7, at room temperature. A platinum foil, Ag/AgCl (saturated KCl), and prepared electrodes PbO_2_/GNR@SS, SnO_2_/MWCNT@SS, and MWCNT@SS were used as the counter, reference, and working electrode, respectively. The measurements were conducted between 0.0 and 3.0 V (vs. Ag/AgCl) at a scanning rate of 10 mV·s^−1^ in 0.1 M Na_2_SO_4_.

### 4.4. Rhodamine B Degradation

The Rhodamine B, with molecular formula C_28_H_31_ClN_2_O_3_ and purity of ≥97.0%, was purchased from Sigma Aldrich (owned by Merck KgaA, Burlington, MA, USA). The electrochemical degradation of Rhodamine B was studied through chronopotentiometric measurements, performed on Gamry Instrument—Interface 1000 Potentiostat/Galvanostat/ZRA06230 (Gamry Instruments, Warminster, PA, USA). A solution of Rhodamine B, concentration 1.14·10^−4^ M in 0.1 M Na_2_SO_4_, was electrolyzed at room temperature in a two-electrode electrolytic cell: MWCNT@SS, SnO_2_/MWCNT@SS, and PbO_2_/GNR@SS were used as working electrodes, and SS electrode was used as a counter electrode. The surface area of the electrodes was 2 cm^2^ (1 × 2 cm), and the applied current density was constant, 20 mA·cm^−2^.

During the decolorization, 12 aliquots of electrolyte were sampled at time intervals from 0 to 30 min. All aliquots were 10 times diluted in distilled water prior to spectrophotometric measurements, to assure the applicability of the Lambert-Beer law. The absorption spectra were recorded in the range of 300–700 nm, on UV/Vis spectrophotometer Lambda 35 (Perkin Elmer, Waltham, MA, USA). The maximum absorbance peak (at 554.5 nm) was used to track the Rhodamine B decolorization.

### 4.5. DFT Calculations

**Figure 10:**
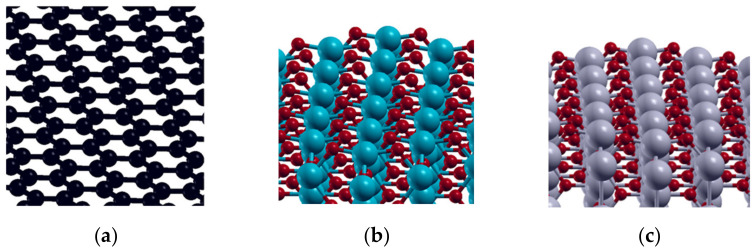
DFT models of investigated surfaces: and: (**a**) graphene (0001); (**b**) SnO_2_-rutile(001) and (**c**) PbO_2_-rutile(001).

A *pwscf* code of the Quantum ESPRESSO package [44] was used to perform DFT calculations within the GGA-PBE approximation [45]. Ultrasoft pseudopotentials (USPP) were used to describe non-valence electrons. The plane-wave cutoff energy was set to 40 eV, while the charge density cutoff was 400 eV. A 3 × 3 (32-atom) graphene supercell was used to represent the basis of the investigated carbonaceous materials (MWCNT and GNR), while 2 × 2 (48-atom cell) was used to describe PbO_2_ and SnO_2_ (Figure 10). Adsorption of OER intermediates (O, OH, and OOH) was investigated on the (0001) plane of graphene and (001) planes of SnO_2_ and PbO_2_.

Optimized lattice parameters were: graphene (*a* = 2.47 A) PbO_2_-rutile (*a* = *c* = 4.99 Å, c = 3.63 Å), and SnO_2_-rutile (*a* = *b* = 4.68 Å, *c* = 3.22 Å).

The distance between slabs was 25 Å, assuring that the vacuum layer above the adsorbed Rhodamine B molecule is thick enough to avoid interaction between periodic images. The graphene surface was fully geometrically optimized prior to and during the adsorption of the OER intermediates, while in the case of PbO_2_ and SnO_2_ (4-layer slabs) two bottom layers were fixed and two top layers were allowed to relax. Adsorption calculations were performed until the residual forces were <0.005 Ry/Bohr. The structural optimization was performed using the Monkhorst-Pack k-point grid (4 × 4 × 4 k-points in bulk optimization and 2 × 2 × 1 k-points in slab calculations of PbO_2_ and SnO_2_). Gaussian smearing was applied to improve convergence. Semiempirical dispersion interaction correction was introduced through the model of Grimme (PBE + D3) [46] as implemented in Quantum ESPRESSO. Isolated molecules: OH, O, and H_2_O were optimized separately, using spin-polarized calculations in a 20 Å × 20 Å × 20 Å supercell and the Martyna-Tuckermann correction for isolated molecules [47]. All input adsorption geometries were *on-top* sites, and the adsorbate molecules were allowed to fully relax during the adsorption. *Bader* code [30] was used to calculate atomic charges. XcrySDen software [48] was used for the graphical representation. Adsorption energies of O and OH (ΔE_ads_) were calculated as the total energy difference between the optimized slab with adsorbate (E_slab+ads_), and the sum of total energies of the isolated molecule (E_tot,isol_) and bare slab (graphene, SnO_2,_ or PbO_2_) (E_tot,slab_).

ΔE_ads_ = E_slab+ads_ − E_tot,slab_ − E_tot,isol_ (7)

Such defined, the more negative ΔE_ads_ means the stronger interaction (stronger adsorption).

Gibbs free energies (at potential E = 0 V vs. RHE) were calculated from the difference in total DFT energies of products and reactants, following the equation:

ΔG_i_ = ΔE_i_ + ΔZPE_i_ − TΔS_i_ (8)

where ΔE_i_ = ∑E_tot, products_ − ∑E_tot_, _reactants_ is the total change of DFT calculated internal energies upon reaction i (i = 1 for OH-formation and i = 2 for OH-deprotonation). ZPE (zero point energy) and TS (entropy term) corrections were taken from [49]. At equilibrium potential of RHE (E = 0 V) free energy of (H^+^ + e^−^) is replaced with the free energy of ½ H_2_.

## Figures and Tables

**Table 1:** Degradation efficiency and apparent rate constants for the investigated electrodes.

Material	Efficiency for 180 min	k_app_ (min^−1^)
MWCNT	41%	0.00288
SnO_2_/MWCNT	73%	0.00653
PbO_2_/GNR	68%	0.00593

**Table 2:** Adsorption energies of OH and atomic O on model surfaces graphene(0001), PbO_2_(001), and SnO_2_(001).

	OH	O
**graphene**	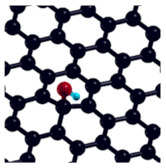	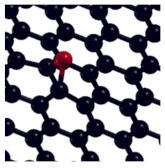
	−0.94 eV	−2.07 eV
**PbO_2_**	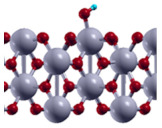	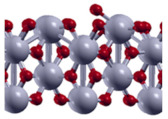
	−1.35 eV	−0.80 eV
**SnO_2_**	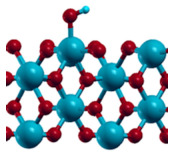	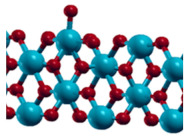
	−1.83 eV	−0.83 eV

## Data Availability

Not applicable.

## Supplementary Materials

The following supporting information can be downloaded at: https://www.mdpi.com/article/10.3390/molecules27165203/s1, Table S1. Atomic percents measured by XPS; Table S2. Fukui indices of Rhodamine B molecule; Figure S1. TEM micrograph of bare SnO_2_ powder.

## Sample Availability

Samples of the compounds SnO_2_/MWCNT and PbO_2_/GNR are available from the authors.
