# Supplementary material for: Is It Possible to Restrain OER on Simple Carbon Electrodes to Efficiently Electrooxidize Organic Pollutants?
Source: Molecules. 2022 Aug 15;27(16):5203. doi: 10.3390/molecules27165203 (PMC9415942; doi:10.3390/molecules27165203)
Supplement: Supplementary file 1 [file molecules-27-05203-s001.zip › molecules-1836873-supplementary.pdf]

# Supplementary Materials

# Is It Possible to Restrain OER on Simple Carbon Electrodes to Efficiently Electrooxidize Organic Pollutants?

**Marija Ječmenica Dučić [1], Danka Aćimović [1], Branislava Savić [1], Lazar Rakočević [2], Marija Simić [1], Tanja Brdarić [1] and Dragana Vasić Anićijević [1,*]**

[1] University of Belgrade, Vinča Institute of Nuclear Sciences-National Institute of the Republic of Serbia, Department of Physical Chemistry, Mike Petrovića Alasa 12–14, 11001 Belgrade, Serbia
[2] University of Belgrade, Vinča Institute of Nuclear Sciences-National Institute of the Republic of Serbia, Department of Atomics Physics, Mike Petrovića Alasa 12–14, 11001 Belgrade, Serbia
* Correspondence: draganav@vin.bg.ac.rs; Tel.: +381-11-34-08-287

**Table S1.** Atomic percents measured by XPS.

| **CNT Pb** | | |
|---|---|---|
| C 1s | 82.9 | % |
| O 1s | 11.1 | % |
| **Pb 4f** | 6 | % |
| CNT Sn | | |
| C 1s | 93.8 | % |
| O 1s | 5.1 | % |
| Sn 3d | 1.1 | % |
| **CNT** | | |
| C 1s | 98.7 | % |
| O 1s | 1.3 | % |

**Table S2.** Fukui indices of Rhodamine B molecule.

| | Nucleophilicity | | | | Radical Attack | | | |
|---|---|---|---|---|---|---|---|---|
| | RhB Atom | q(N) | q(N − 1) | Fa- | Rhb Atom | q(N + 1) | q(N − 1) | Fa0 |
| 1 | O | 7.857322 | 7.74038 | 0.116942 | O | 7.906223 | 7.74038 | 0.165843 |
| 2 | O | 7.670466 | 7.421019 | 0.249447 | O | 7.747194 | 7.421019 | 0.326175 |
| 3 | O | 7.637462 | 7.626688 | 0.010774 | O | 7.645222 | 7.626688 | 0.018534 |
| 4 | N | 7.446052 | 7.294789 | 0.151263 | N | 7.499211 | 7.294789 | 0.204422 |
| 5 | N | 6.975949 | 7.485839 | −0.50989 | N | 6.930749 | 7.485839 | −0.55509 |
| 6 | C | 1.132712 | 1.237361 | −0.10465 | C | 1.126596 | 1.237361 | −0.11077 |
| 7 | C | 4.094932 | 4.064295 | 0.030637 | C | 4.103981 | 4.064295 | 0.039686 |
| 8 | C | 4.071462 | 4.054048 | 0.017414 | C | 4.087168 | 4.054048 | 0.03312 |
| 9 | C | 3.998633 | 3.999706 | −0.00107 | C | 4.011458 | 3.999706 | 0.011752 |
| 10 | C | 4.085829 | 4.087221 | −0.00139 | C | 4.090176 | 4.087221 | 0.002955 |
| 11 | C | 4.007695 | 4.0054 | 0.002295 | C | 4.011354 | 4.0054 | 0.005954 |
| 12 | C | 4.046196 | 4.044938 | 0.001258 | C | 4.079576 | 4.044938 | 0.034638 |
| 13 | C | 4.071093 | 4.069466 | 0.001627 | C | 4.073752 | 4.069466 | 0.004286 |
| 14 | C | 4.018033 | 4.043469 | −0.02544 | C | 4.023992 | 4.043469 | −0.01948 |
| 15 | C | 4.059778 | 4.01796 | 0.041818 | C | 4.056647 | 4.01796 | 0.038687 |
| 16 | C | 3.260852 | 3.320457 | −0.05961 | C | 3.215158 | 3.320457 | −0.1053 |
| 17 | C | 3.217452 | 3.298414 | −0.08096 | C | 3.179920 | 3.298414 | −0.11849 |
| 18 | C | 3.677878 | 3.427324 | 0.250554 | C | 3.643711 | 3.427324 | 0.216387 |
| 19 | C | 3.691415 | 3.438835 | 0.25258 | C | 3.732133 | 3.438835 | 0.293298 |
| 20 | C | 4.054983 | 4.039877 | 0.015106 | C | 4.074325 | 4.039877 | 0.034448 |
| 21 | C | 4.079402 | 4.065299 | 0.014103 | C | 4.090166 | 4.065299 | 0.024867 |
| 22 | C | 3.955236 | 3.953486 | 0.00175 | C | 3.979989 | 3.953486 | 0.026503 |
| 23 | C | 4.000374 | 3.999982 | 0.000392 | C | 4.039235 | 3.999982 | 0.039253 |
| 24 | C | 4.01917 | 3.992409 | 0.026761 | C | 4.041488 | 3.992409 | 0.049079 |
| 25 | C | 2.479919 | 2.487303 | −0.00738 | C | 2.470000 | 2.487303 | −0.0173 |
| 26 | C | 4.020836 | 4.014758 | 0.006078 | C | 3.999273 | 4.014758 | −0.01548 |
| 27 | C | 3.030975 | 3.021861 | 0.009114 | C | 3.092706 | 3.021861 | 0.070845 |
| 28 | C | 2.631027 | 2.604319 | 0.026708 | C | 2.691442 | 2.604319 | 0.087123 |
| 29 | C | 3.326335 | 3.304751 | 0.021584 | C | 3.325081 | 3.304751 | 0.02033 |
| 30 | C | 4.787271 | 4.770424 | 0.016847 | C | 4.780000 | 4.770424 | 0.009576 |
| 31 | C | 3.970301 | 3.964803 | 0.005498 | C | 3.990442 | 3.964803 | 0.025639 |
| 32 | C | 4.019869 | 4.015367 | 0.004502 | C | 4.017893 | 4.015367 | 0.002526 |
| 33 | C | 3.958548 | 3.956734 | 0.001814 | C | 3.995756 | 3.956734 | 0.039022 |
| 34 | H | 0.92037 | 0.89109 | 0.02928 | H | 0.952720 | 0.89109 | 0.06163 |
| 35 | H | 0.963422 | 0.928827 | 0.034595 | H | 0.994867 | 0.928827 | 0.06604 |
| 36 | H | 0.874841 | 0.862776 | 0.012065 | H | 0.893311 | 0.862776 | 0.030535 |
| 37 | H | 0.950257 | 0.926371 | 0.023886 | H | 0.970004 | 0.926371 | 0.043633 |
| 38 | H | 0.972138 | 0.968097 | 0.004041 | H | 0.970997 | 0.968097 | 0.0029 |
| 39 | H | 0.958419 | 0.942727 | 0.015692 | H | 0.969153 | 0.942727 | 0.026426 |
| 40 | H | 1.006117 | 0.983609 | 0.022508 | H | 1.022024 | 0.983609 | 0.038415 |
| 41 | H | 0.973461 | 0.973067 | 0.000394 | H | 0.971908 | 0.973067 | −0.00116 |
| 42 | H | 0.995152 | 0.980892 | 0.01426 | H | 1.005080 | 0.980892 | 0.024188 |
| 43 | H | 0.97336 | 0.959972 | 0.013388 | H | 0.997111 | 0.959972 | 0.037139 |
| 44 | H | 0.982663 | 0.972234 | 0.010429 | H | 0.997771 | 0.972234 | 0.025537 |
| 45 | H | 0.924575 | 0.924899 | −0.00032 | H | 0.924829 | 0.924899 | $-7 \times 10^{-5}$ |
| 46 | H | 0.932392 | 0.914566 | 0.017826 | H | 0.956409 | 0.914566 | 0.041843 |
| 47 | H | 0.984957 | 0.974993 | 0.009964 | H | 0.997832 | 0.974993 | 0.022839 |
| 48 | H | 0.954584 | 0.953993 | 0.000591 | H | 0.956705 | 0.953993 | 0.002712 |
| 49 | H | 0.983982 | 0.925903 | 0.058079 | H | 0.995835 | 0.925903 | 0.069932 |
| 50 | H | 0.976985 | 0.952641 | 0.024344 | H | 0.995835 | 0.952641 | 0.043194 |
| 51 | H | 0.96807 | 0.953441 | 0.014629 | H | 0.975736 | 0.953441 | 0.022295 |
| 52 | H | 0.973857 | 0.950756 | 0.023101 | H | 0.988661 | 0.950756 | 0.037905 |
| 53 | H | 0.991473 | 0.976716 | 0.014757 | H | 1.001558 | 0.976716 | 0.024842 |
| 54 | H | 0.947746 | 0.940559 | 0.007187 | H | 0.961080 | 0.940559 | 0.020521 |
| 55 | H | 0.971623 | 0.95299 | 0.018633 | H | 0.997012 | 0.95299 | 0.044022 |
| 56 | H | 0.952402 | 0.946078 | 0.006324 | H | 0.964061 | 0.946078 | 0.017983 |
| 57 | H | 0.929275 | 0.91518 | 0.014095 | H | 0.952839 | 0.91518 | 0.037659 |
| 58 | H | 0.950161 | 0.919396 | 0.030765 | H | 0.980346 | 0.919396 | 0.06095 |
| 59 | H | 0.93309 | 0.904368 | 0.028722 | H | 0.970795 | 0.904368 | 0.066427 |

| | | | | | | | | |
|---|---|---|---|---|---|---|---|---|
| 60 | H | 0.933681 | 0.92346 | 0.010221 | H | 0.953662 | 0.92346 | 0.030202 |
| 61 | H | 0.918723 | 0.911726 | 0.006997 | H | 0.948127 | 0.911726 | 0.036401 |
| 62 | H | 0.938901 | 0.917584 | 0.021317 | H | 0.959243 | 0.917584 | 0.041659 |
| 63 | H | 0.907826 | 0.882045 | 0.025781 | H | 0.943425 | 0.882045 | 0.06138 |

C-atoms 18 and 19, which are identified as the most reactive carbon atoms according to the indices, belong to ethyl groups bound to N – heteroatoms. **NOTE:** Although reactive, oxygen heteroatoms were not taken into consideration in cleavage site prediction, since their reactivity does not contribute to the cleavage of carbon chain.

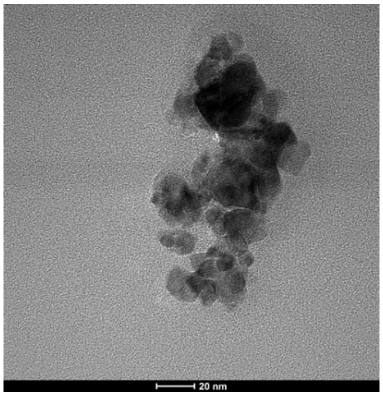


**Figure S1.** TEM micrograph of bare $SnO_2$ powder.
